# A Rare Case of Cutis Verticis Gyrata with Underlying Cerebriform Intradermal Nevus

**DOI:** 10.7759/cureus.6499

**Published:** 2019-12-29

**Authors:** Lisa F Fronek, Kate Braunlich, Maheera Farsi, Richard A Miller

**Affiliations:** 1 Dermatology, Hospital Corporation of America/University of South Florida, Morsani College of Medicine, Largo Medical Center Program, Largo, USA; 2 Dermatology, Largo Medical Center, Largo, USA

**Keywords:** cutis verticis gyrata, cerebriform intradermal nevus, scalp, dermatology, melanoma

## Abstract

Cutis verticis gyrata (CVG) is an uncommon condition of the scalp known for redundant, thickened folds, which emulate the cerebral gyri of the brain. This unusual finding is catalogued as primary essential, primary non-essential, and secondary. While primary essential CVG is an isolated and idiopathic condition, primary non-essential CVG is deemed to be related to neurological, ophthalmological, or psychiatric disorders. Secondary CVG may be due to a variety of systemic disorders, inflammatory dermatoses, or cutaneous neoplasms or infiltrates. This report serves as an example of secondary CVG due to a cerebriform intradermal nevus, with specific focus on clinical course, treatment options, and critical screening guidelines for these patients.

## Introduction

Cutis verticis gyrata (CVG) is a rare scalp disorder manifested by scalp skin redundancy that mirrors the folds of cerebral gyri. While the worldwide prevalence is currently undetermined due to the rarity of the condition, the estimated prevalence is 1 in 100,000 in the male population and 0.026 in 100,000 in the female population [1]. This condition may be inherited or acquired, and is further categorized into primary essential, primary non-essential, and secondary CVG depending on the etiology. There is no ethnic predilection, and this condition can occur at any age. At this point there is no single pathogenesis for this disorder; however, there are many systemic diseases which may be found in association with CVG. Primary essential CVG is an isolated finding with no association with any other conditions. Primary non-essential CVG has been linked to neuropsychiatric or ophthalmological disorders. Secondary CVG occurs in patients with underlying systemic diseases, inflammatory dermatoses, trauma, or benign skin tumors, such as cerebriform intradermal nevus (CIN), neurofibroma, dermatofibroma, or collagenoma [1]. CIN is a rare cause of secondary CVG, representing 12.5% of all CVG cases [2]. The diagnosis of CIN is both clinical and histopathological, and it typically presents as an asymmetric growth of the parietal or occipital scalp [3]. CIN has a female predominance and characteristically starts at birth or childhood as an asymptomatic, skin-colored macule that slowly enlarges throughout the patient’s life [4]. The most striking concern when treating and monitoring these patients is the small but notable risk of development of malignant melanoma within a CIN [3,5,6]. The purpose of this case report is to present an unusual case of a secondary CVG in a 46-year-old female with an underlying CIN, and to discuss the clinical course, treatment options, and appropriate screening for melanoma within this lesion.

## Case presentation

**Figure 1:**
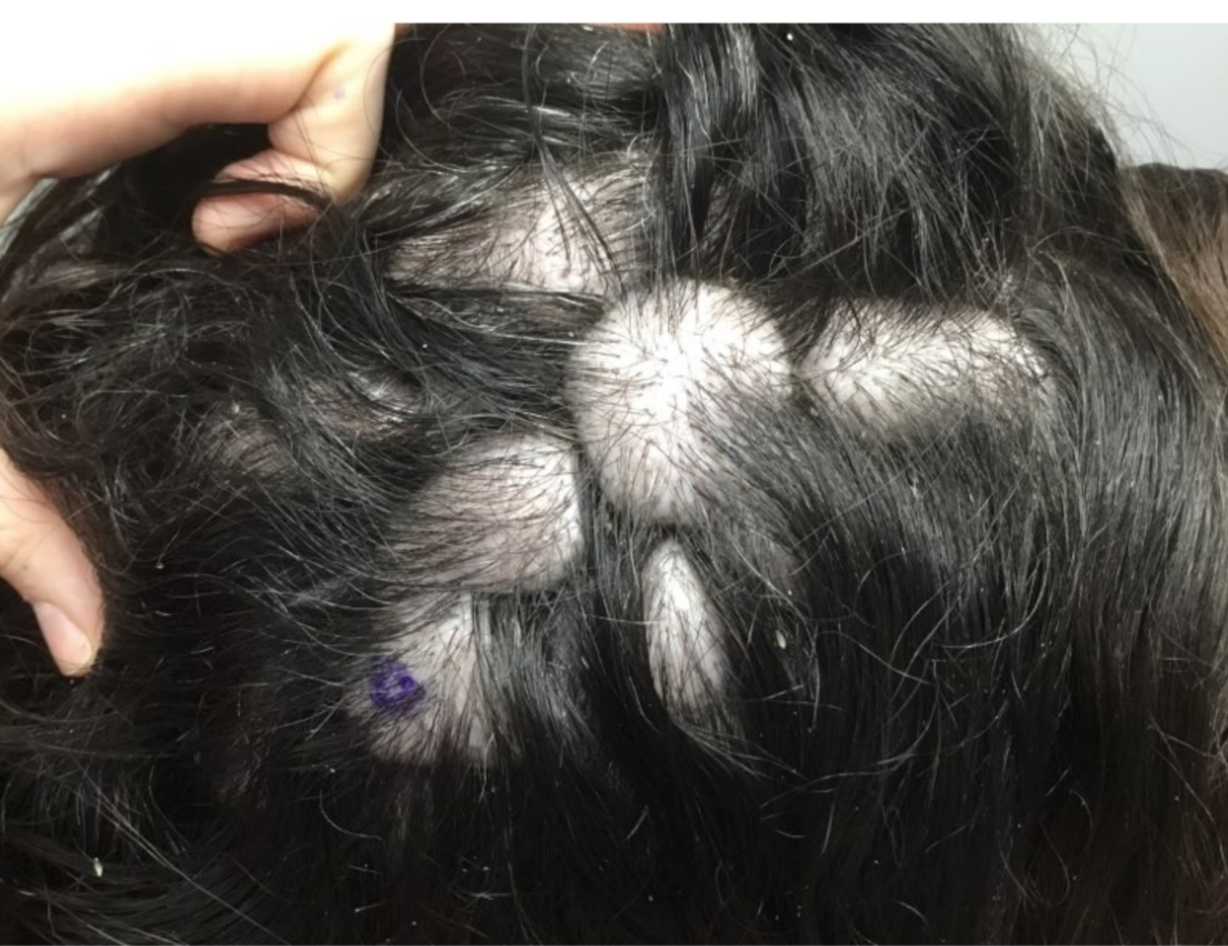
Prominent skin folds were noted on the right superior parietal scalp, left superior parietal scalp, right superior occipital scalp, and left superior occipital scalp with co-existing diffuse non-scarring hair loss distributed.

**Figure 2:**
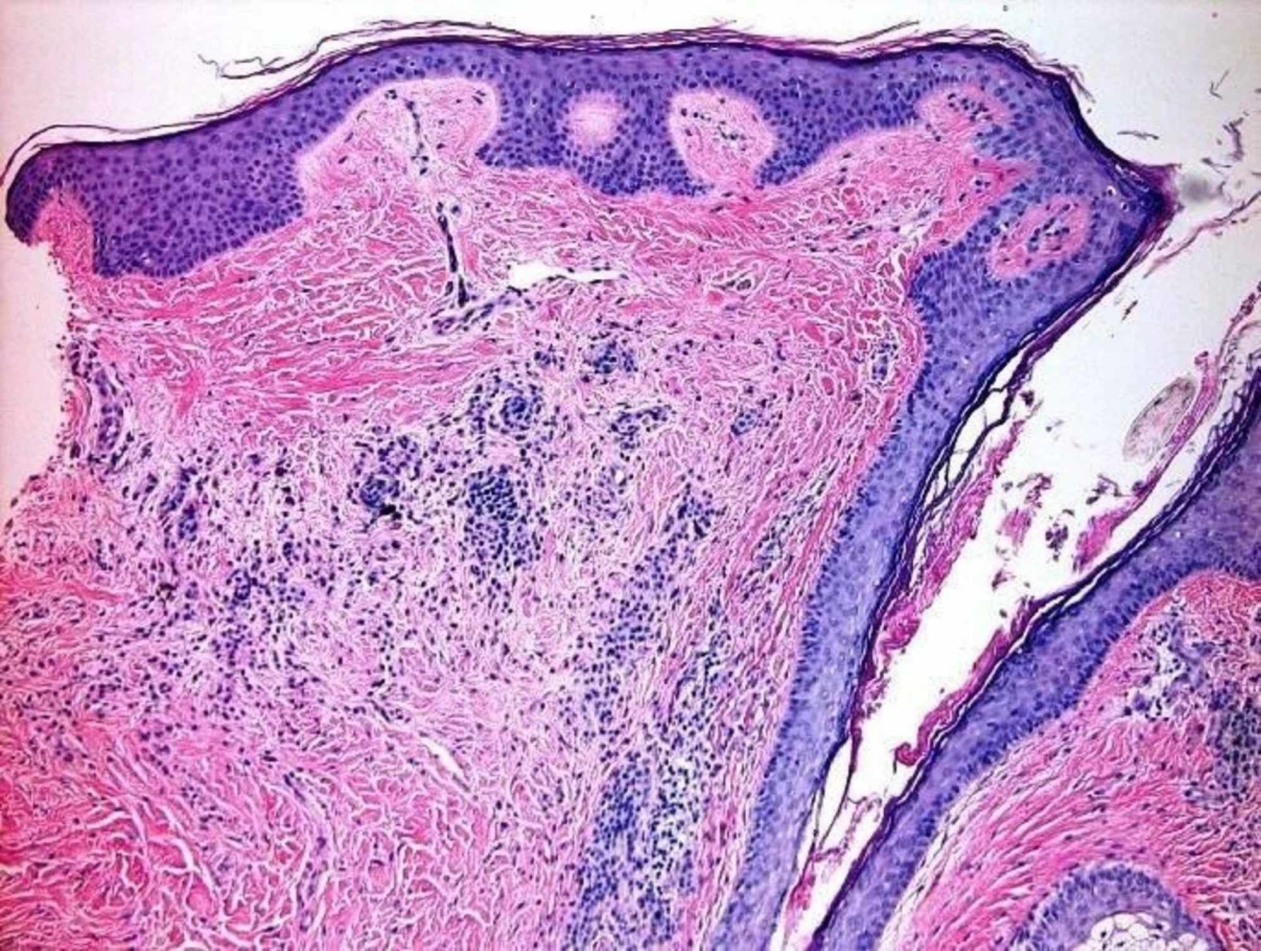
One 6.0 mm punch biopsy of the right superior occipital scalp was obtained for H&E, which showed subtle, non-scarring, non-inflammatory alopecia; this could represent telogen effluvium. The absence of significant miniaturization is evidence against androgenic alopecia. H&E, hematoxylin and eosin.

**Figure 3:**
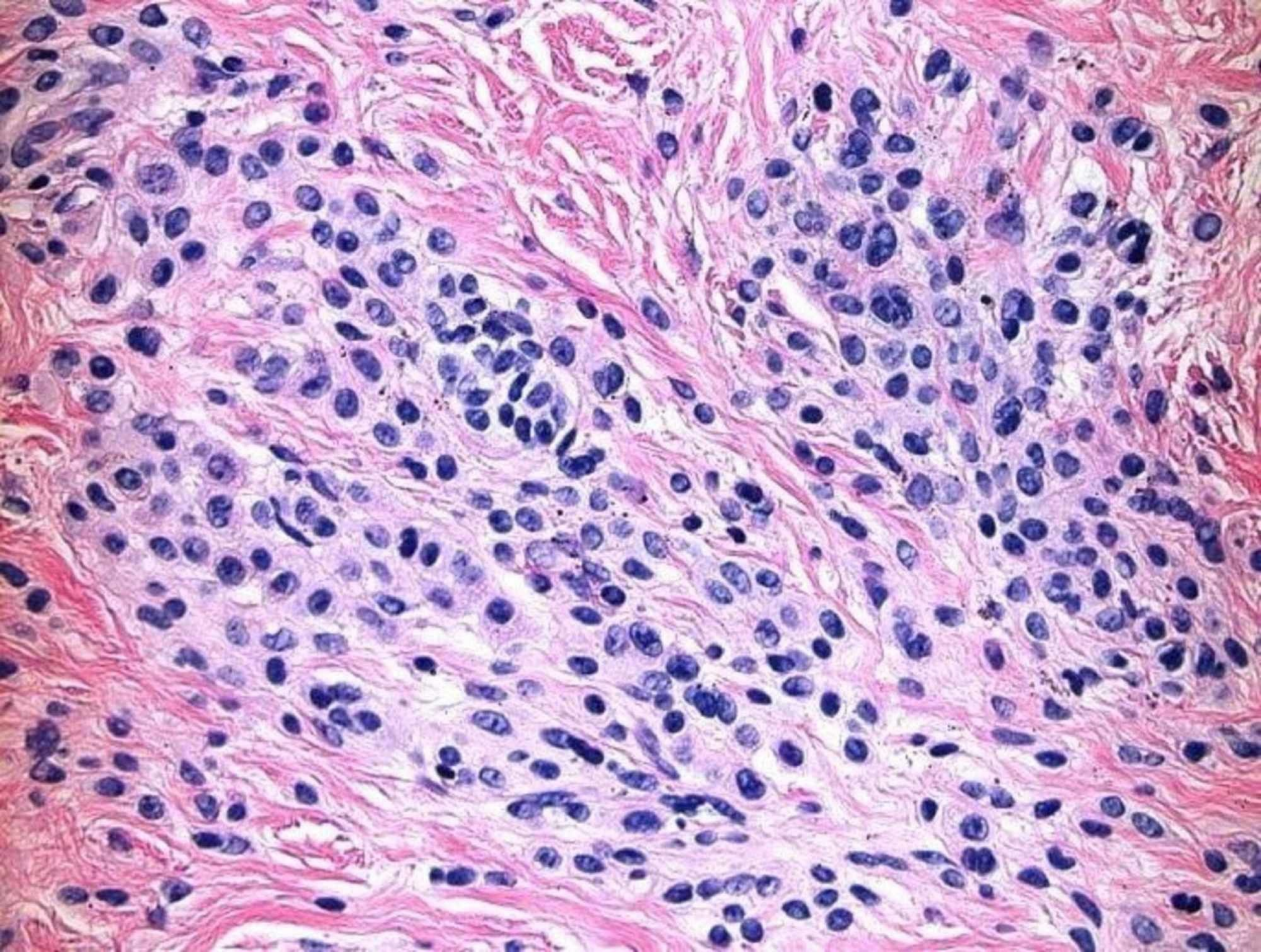
There is background intradermal melanocytic nevus with congenital features. Small, round, type B melanocytes present in nests in the reticular dermis. Type C melanocytes present deeper in the reticular dermis forming into regular nests. No atypia identified. No features of a deposition disorder identified.

A 46-year-old Caucasian female presented with a chief complaint of generalized hair loss for a duration of nine months. The patient stated that the hair loss was gradual in onset and had worsened over time. She had no neurological or ophthalmological complaints to report. Her past medical history was significant for Graves' disease and atrial fibrillation. There was no history of inflammatory skin disorders. Surgical history was pertinent for a pacemaker placement, salivary gland excision, and Achilles tendon repair. Family history was unremarkable. She was a former smoker and alcohol intake noted to be less than one drink daily. She denied any illicit drug use. She had allergies to shellfish-derived foods; however, no known drug allergies. Medications included venlafaxine, buprenorphine/naloxone, levothyroxine, metoprolol succinate, trazodone, and alprazolam. Her review of systems was otherwise unremarkable, specifically negative for weight loss, night sweats, and fatigue. On clinical physical exam, the patient appeared healthy and was in no acute distress; vital signs were within normal limits. Prominent skin folds were noted on the right superior parietal scalp, left superior parietal scalp, right superior occipital scalp, and left superior occipital scalp (Figure 1). Additionally, there was diffuse non-scarring hair loss distributed on the posterior mid-parietal scalp and right superior occipital scalp. Complete blood count (CBC), basic metabolic profile BMP), thyroid-stimulating hormone (TSH), iron, total iron-binding capacity (TIBC), dihydrotestosterone (free, serum), testosterone (free and total), and anti-nuclear antibody multiplex (ANA) with reflex were ordered. Of note, this patient was not referred for brain magnetic resonance imaging (MRI) due to the lack of neurological or psychiatric findings. One 6.0 mm punch biopsy of the right superior occipital scalp was obtained for hematoxylin and eosin stain, which showed subtle, non-scarring, non-inflammatory alopecia, representative of telogen effluvium (Figure 2). No features of a deposition disorder were identified; however, there is background intradermal melanocytic nevus with congenital features (Figure 3). The patient described above was treated symptomatically with clobetasol 0.05% scalp solution twice a day for two weeks, biotin 5,000 µg daily, and tricomin shampoo without significant improvement. Additionally, she was counseled regarding surgical treatment options, as well as the literature-based risk of malignant transformation; the patient deferred all surgical options. 

## Discussion

CVG is an infrequent disorder of the scalp characterized by ridges and folds that resemble the structure of the cerebral cortex [7]. The worldwide prevalence of CVG is difficult to establish due to the extreme rarity of this condition. Of the known reports of CVG, there is a definite male predilection, with an estimated prevalence of 1 in 100,000 in the male population and 0.026 in 100,000 in the female population [1]. CVG can occur at any age and in either sex; however, there are several reports of primary essential CVG presenting in pre-pubertal men. CVG most commonly affects the vertex and occiput; it may occasionally affect the complete scalp [8]. There are typically 2-30 folds that follow a symmetric pattern in an anterior to posterior direction [8]. While there is no known etiology and the pathogenesis remains undefined, several disorders are associated with this condition, as described below.

CVG is classified into three categories depending on the intrinsic etiology: primary essential, primary non-essential, and secondary. Primary essential CVG is a solitary finding, with no association with any neurological, endocrine, or ophthalmological disease [9]. Many patients are asymptomatic, and they present for evaluation due to cosmetic reasons. Other symptoms include pruritus, generalized headaches, or diffuse hair loss, as seen in this case [8,10]. It has been postulated that primary essential CVG may be hormonally driven, as many of the case reports demonstrate a male post-pubertal presentation; however, no hormonal alteration has successfully been correlated [9,10]. Primary non-essential CVG is typically affiliated with underlying neuropsychiatric disorders, such as seizures, mental retardation, and other brain or ophthalmological conditions [11]. Between 0.2% and 4.5% of patients with primary non-essential CVG have concomitant intellectual disability [12]. Finally, secondary CVG can be related to a variety of other disorders; these include hormonally driven diseases such as acromegaly, thyroid conditions, and myxedema [13]; hematologic diseases such as leukemia or amyloidosis [14,15]; infectious agents such as syphilis or human immunodeficiency virus (HIV) [16]; inflammatory skin disorders such as eczema, psoriasis, or Darier’s disease [17]; solitary neoplasms; and genodermatoses such as Noonan syndrome, tuberous sclerosis complex, or neurofibromatosis [7,15]. CIN is one of several benign skin tumors to cause CVG, and overall accounts for 12.5% of CVG cases [2]. Orkin et al. demonstrated that patients with CIN causing CVG have a normal intellectual ability, there is a slight female predominance, and there is no association with local or systemic diseases. The presenting symptom for this condition varies and depends primarily on the underlying disease pathology. In the case we are presenting, we ordered prompt laboratory testing that included CBC, BMP, TSH, iron and TIBC, dihydrotestosterone (free, serum), testosterone (free and total), and ANA multiplex with reflex. The only positive finding was a slightly elevated centromere B antibody, which may be present in 27% systemic sclerosis, 66% CREST syndrome, 3%-12% systemic lupus erythematosus, 7% mixed connective tissue disease, and <2% Sjogren’s syndrome, polymyositis, and normal blood donors. Certain groups chose to expand this work-up to offer testing for immunoglobulins, growth hormone, insulin growth factor-1, insulin, insulin resistance index, hepatitis, HIV, syphilis, karyotype, plain radiographs, and brain MRI [10]. On histopathological examination, there is a range from normal findings of the epidermal and dermal structures, to the appearance of increased collagenization with hypertrophy or hyperplasia of the surrounding adnexa [1]. Occasionally, there may be co-existence of an increased number of collagen fibers that may encompass nearby apocrine and eccrine glands [9,18]. Additionally, in secondary CVG there may be additional histopathological findings of the root dermatologic disease process. The differential diagnosis for CVG includes dissecting cellulitis of the scalp, focal acanthosis nigricans of the neck, and CIN. Management of primary essential CVG emphasizes local skin care and scalp hygiene, as it is a benign disease and has no associated diseases or risk of malignancy. It would behoove providers to refer patients with primary non-essential and secondary CVG to the appropriate specialists, such as ophthalmologists, neurologists, psychiatrists, or hematologists, depending on the patient’s presenting symptoms and underlying diagnosis. Treatment of secondary CVG should emphasize targeting the underlying medical disease. Patient with all three types of CVG may desire surgical excision of the overlapping skin folds to provide a flat and smooth cosmetic appearance [8,19].

This is a case of secondary CVG with CIN. While our patient did have a history of Graves’ disease, she had been successfully treated with thyroid replacement, as demonstrated by her markedly normal TSH (0.69 mIU/L, range 0.40-4.50 mIU/L). Our patient was treated with clobetasol 0.05% solution, biotin 5,000 µg daily, and tricomin shampoo. She was counseled regarding treatment options, including surgical resection and reconstruction. For patients in which the aesthetic aspect of CVG is their main complaint, surgical excision is the primary treatment goal [18]. In addition to the above patient education and counseling, arguably the most convincing reason for surgical excision of CIN is the associated 4.5% risk of a malignant melanoma developing within the CIN [3,5]. Within dermatologists who have observed these findings, there is no agreed upon course of action; some opt for aggressive surgical excision, whereas other providers deem close clinical follow-up is sufficient [20]. The patient of this case was educated about the 4.5% risk of malignant melanoma developing within her CIN, and she deferred any further evaluation and treatment. 

## Conclusions

The above documents a case of secondary CVG due to an underlying CIN that was incidentally found. These patients typically seek treatment due to alopecia of the scalp and cosmetic concerns. Additionally, pruritus, dysesthesia, tenderness, pain, and increased risk of local infections may co-exist with this form of CVG. There is currently debate whether surgical excision is unequivocally required, or close follow-up is sufficient given the low rate of malignant change and the extensive surgical margins involved with CVG. The goal of this case report is to discuss a rare dermatologic diagnosis, and the small but present risk of developing malignant melanoma within a CIN. 
